# LC-MS/MS-Based Proteomics Approach for the Identification of Candidate Serum Biomarkers in Patients with Narcolepsy Type 1

**DOI:** 10.3390/biom13030420

**Published:** 2023-02-23

**Authors:** Akeem Sanni, Mona Goli, Jingfu Zhao, Junyao Wang, Chloe Barsa, Samer El Hayek, Farid Talih, Bartolo Lanuzza, Firas Kobeissy, Giuseppe Plazzi, Monica Moresco, Stefania Mondello, Raffaele Ferri, Yehia Mechref

**Affiliations:** 1 Chemistry and Biochemistry Department, Texas Tech University, Lubbock, TX 79409, USA; 2 Faculty of Biochemistry and Molecular Genetics, American University of Beirut, Beirut 1107 2020, Lebanon; 3 Department of Psychiatry and Behavioral Sciences, University of Miami Miller School of Medicine, Miami, FL 33124, USA; 4 Department of Psychiatry, Faculty of Medicine, American University of Beirut, Beirut 1107 2020, Lebanon; 5 Sleep Research Centre, Department of Neurology IC, Oasi Research Institute-IRCCS, 94018 Troina, Italy; 6 Multiomics & Biomarkers, Department of Neurobiology, Center for Neurotrauma, Morehouse School of Medicine (MSM), Atlanta, GA 30310, USA; 7 IRCCS, Instituto delle Scienze Neurologiche di Bologna, 40139 Bologna, Italy; 8 Department of Biomedical, Metabolic and Neural Sciences, University of Modena and Reggio Emilia, 41125 Modena, Italy; 9 Department of Biomedical and Dental Sciences and Morphofunctional Imaging, University of Messina, 98122 Messina, Italy

**Keywords:** autoimmunity, NT1, pathophysiology, serum-protein biomarkers, orexin/hypocretin

## Abstract

Narcolepsy type 1 (NT1) is the most common type of narcolepsy known to be caused by the loss of specific neurons responsible for producing peptide neurotransmitters (orexins/hypocretins), resulting in a sleep-wake cycle disorder. It is characterized by its association with cataplexy and abnormalities in rapid eye movement. To date, no cure has been established for this life-threatening condition. Misdiagnosis of NT1 is also quite common, although it is not exceedingly rare. Therefore, successfully identifying candidate serum biomarkers for NT1 would be a head start for accurate diagnosis and development of therapeutics for this disorder. This study aims to identify such potential serum biomarkers. A depletion protocol was employed for 27 human serum samples (16 NT1 and 11 healthy controls), followed by applying LC-MS/MS bottom-up proteomics analysis, then LC-PRM-MS for validation. The comparison of the proteome profiles of the low-abundant proteins in the samples was then investigated based on age, sex, sample groups, and the presence of the Human Leukocyte Antigen (HLA) DQB1*0602 allele. The results were tracked to gene expression studies as well as system biology to identify key proteins and understand their relationship in the pathogenesis of NT1. Our results revealed 36 proteins significantly and differentially expressed. Among the impaired pathways and bioprocesses, the complement activation pathway is impaired by six of the differentially expressed proteins (DEPs). They are coded by the genes C2, CFB, C5, C1R, C1S, and MASP1, while 11 DEPs are involved in Acute Phase Response Signaling (APRS), which are coded by the genes FN1, AMBP, APOH, CFB, CP, ITIH2, C5, C2, F2, C1, and ITIH4. The combined AUCs of the downregulated and upregulated DEPs are 0.95 and 0.76, respectively. Overall, this study reveals potential serum-protein biomarkers of NT1 and explains the possible correlation between the biomarkers and pathophysiological effects, as well as important biochemical pathways involved in NT1.

## 1. Introduction

Narcolepsy type 1 (NT1) is a sleep disorder associated with cataplexy, a neurological condition characterized by muscle paralysis and decreased sensitivity to pain [1]. The disease is characterized by a high level of loss or dysfunction of orexin/hypocretin neurons in the lateral region of the hypothalamus without affecting the melanin-concentrating hormone neurons [2]. Apart from sleep-wake regulation, it is important to state that the dysfunction of the orexinergic system affects other metabolic processes and complicates some metabolic diseases such as type-2 diabetes and obesity [3,4]. In the 1950s, excessive daytime sleepiness and sleep paralysis, hypersomnia, and cataplexy were considered the major symptoms of narcolepsy [5]. Over time, the correct and scientific diagnosis of NT1 became more complicated and not limited to these symptoms. Christian and Claudio, in 2004, compared the two types of narcolepsy and attempted to differentiate their common cataplexy-like symptoms from ‘true’ cataplexy in NT1 [6]. Since NT1 comes with cataplexy-like symptoms, it differs from narcolepsy without definite cataplexy, and it is also the most common type of narcolepsy [7]. The existence of co-occurring symptoms proves the scientific need to investigate and identify biomarkers for NT1; this would improve the accurate diagnosis and categorization of this disease.

On the other hand, proteome profiling is one of the vital experimental studies for biomarker discovery in biological and biomedical samples [8]. Using this method, the proteome of a particular cell or biofluid is identified, and the proteins that are differentially expressed are identified and further analyzed using bioinformatics techniques. Therefore, serum-based proteomics gives the advantage of revealing disease progression. Developing techniques for biomarker identification, exploring expression levels of the comprehensively identified proteins, and associating those changes to different disease conditions using bioinformatics software help in the discovery of clinically relevant biomarkers [9,10]. Validation of the data is necessary to factor in the effect of FDR during in-silico digestion of most bioinformatic tools utilized in proteomics studies. Western blotting has often been applied, but to improve analysis throughout, mass spectrometry (MS)-based targeted analysis was developed, and parallel reaction monitoring (PRM) is now commonly used.

MS is a very sensitive analytical tool that takes advantage of its high mass accuracy and resolution for detecting and quantifying analytes [11,12]. MS coupled with other instruments for separation, such as capillary electrophoresis and liquid chromatography, improves the ability of MS to serve a multi-purpose bioanalytical role [13,14]. This leads to an improvement in proteomics studies and the development of biomarkers in the scientific community [15]. Liquid chromatography coupled with tandem mass spectrometry (LC-MS/MS) is a versatile bioanalytical technique for high-throughput quantitative analysis of complex biological samples, especially in proteomics [16,17]. Previously, several studies have considered LC-MS/MS for measurements of orexin-A in narcolepsy and proteomics profiling of the hypothalamus in mouse models of narcolepsy [18,19]. However, to the best of our knowledge, identifying and quantifying potential biomarkers in human serum samples of NT1 has never been done.

In this study, we apply LC-MS/MS techniques for label-free proteomics quantification (LFPQ) and targeted proteomics, followed by system biology, to identify potential biomarkers of human sera samples in NT1. In addition, this study evaluates the correlation between narcolepsy and immunity/autoimmunity, inflammation, and degeneration, as well as other biological, cellular, and molecular processes involved in this disease. Overall, the results of this study will provide information on candidate serum-protein biomarkers for narcolepsy type 1 that could be targeted for accurate serum diagnosis and development of therapeutics for narcolepsy type 1. This should directly and significantly indicate new ways to improve the diagnosis of NT1 in serum samples of patients, as well as assist in the development of potential therapeutics.

## 2. Materials and Methods

### 2.1. Chemicals and Reagents

(HPLC)-grade water (H_2_O), acetonitrile (ACN), and MS-grade formic acid (FA) were obtained from Fisher Scientific (Fair Lawn, NJ, USA). Ammonium bicarbonate (ABC), dithiothreitol (DTT), and iodoacetamide (IAA) were purchased from Sigma-Aldrich (St. Louis, MO, USA). Trypsin/Lys-C mix MS-grade was purchased from Promega (Madison, WI, USA).

### 2.2. Study Participants

Patients with a clinical diagnosis of NT1 were recruited from the Department of Neurology and Sleep Medicine, Oasi Research Institute (IRCCS), and the Department of Neurology, University of Bologna, Italy, between April and June 2018. For patients with NT1, a clinical and laboratory diagnosis of NT1, according to the 3rd edition of the International Classification of Sleep Disorders, was required. It was recommended that the following criteria be met [20]: (a) unequivocal cataplexy during laboratory testing; (b) persistent daytime sleepiness; (c) at least 2 SOREMPs and an 8-min mean sleep latency during the multiple sleep latency tests; (d) evidence of cerebrospinal fluid orexin A deficiency, when available. The presence of human leukocyte antigen (HLA) DQB1*0602 was assessed in all patients. Patients were excluded if they had any other neurologic or medical conditions (including hypertension and diabetes, in particular) and if they were taking any medications. All patients were enrolled soon after the diagnosis was made; for this reason, they were all drug-naïve at the time of the blood sampling, none were obese, and their body mass indexes were not excessive (5 overweight patients out of 16, with values ≥25 <30). None of these patients had Streptococcus infection, H1N1 flue or use of AS03-adjuvanted vaccine prior to the onset of narcoleptic symptoms. Healthy participants served as controls. The study was approved by the local ethics committee. All participants provided written informed consent before participating in the study, and their demographic and some clinical information is presented in Table 1. The serum samples were collected between the hours of 9 AM–12 PM.

### 2.3. Depletion of High-Abundance Proteins in Serum Samples

**Figure 1:**
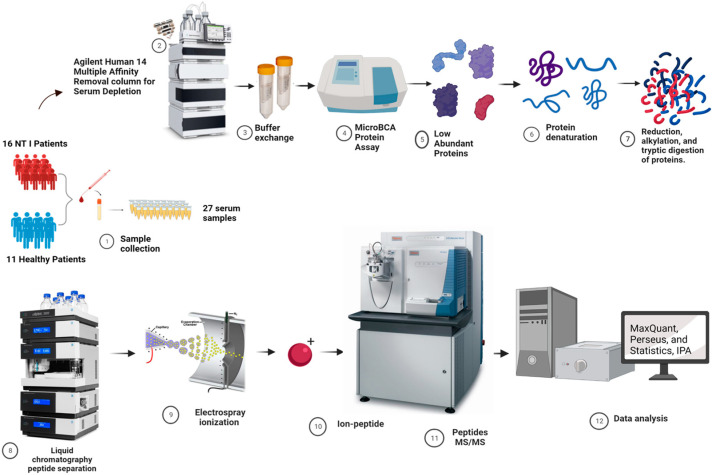
Experimental workflow that summarizes the sample preparation and analysis of the NT1 proteomics experiment. The bottom-up proteomics study starts from sample collection, high-abundant protein depletion processes, tryptic digestion of proteins into peptides, LC-MS/MS analyses of peptides, and data processing.

As seen in Figure 1, the experimental processes followed in this study started with the depletion of the collected serum samples. A total of 27 serum samples, consisting of 11 healthy controls and 16 NT1 samples, were subjected to depletion using Agilent Human 14 multiple affinity removal column with dimension 4.6 × 100 mm. This column depletes the 14 high-abundant proteins, namely albumin, IgA, transferrin, haptoglobin, IgG, antitrypsin, transferrin, alpha 1-acid glycoprotein, IgM, apolipoprotein AI, apolipoprotein AII, transthyretin, complement C3, and alpha 2-macroglobulin. The depletion process helps expand the dynamic range of quantitative and qualitative analysis of the low-abundant proteins that might be differentially expressed as a result of NT1 disease. Therefore, a 30 µL aliquot of serum was depleted following the protocol provided by the manufacturer. After depletion, using 5KDa MWCO 4 mL spin concentrator Agilent filter (Santa Clara, CA, USA), the buffer in the samples was exchanged with 50 mM ammonium bicarbonate (ABC) (pH 8.0) which is more compatible with Mass spectrometry, and was used for tryptic digestion.

### 2.4. Tryptic Digestion

The depleted sera were further prepared based on previously established protocols [21]. The protein concentration of depleted sera samples was determined before tryptic digestion using the micro-BCA protein assay following the protocol recommended by the vendor (Thermo Scientific/Pierce, Rockford, IL, USA). An aliquot of 15 µg of depleted serum proteins corresponding to 0.2 µL of original serum was transferred to an Eppendorf tube, followed by the addition of 50 mM ammonium bicarbonate to have the final volume of 50 µL. Thermal denaturation was performed at 80 °C for 30 min. The denatured proteins were reduced and alkylated with DTT and IAA, respectively [22]. DTT and IAA solutions were prepared in 50 mM ammonium bicarbonate. Initially, 1.25 µL of DTT was added to the samples and incubated at 60 °C for 45 min, then samples were alkylated with 5 µL of IAA solution and incubated at 37.5 °C for 45 min. Subsequently, alkylation was quenched with another 1.25 µL of DTT, and incubation was done at 37.5 °C for 30 min. To digest the reduced and alkylated proteins into peptides, 0.6 µg of trypsin/lysine C was added to the samples and then incubated at 37.5 °C for 18 h. Later, the digestion was terminated by the addition of 0.3 µL of neat FA. The samples were speed-vacuum dried, resuspended in a solution containing 98% HPLC water, 2% ACN, and 0.1% FA, and then subjected to liquid chromatography coupled with tandem mass spectrometry (LC-MS/MS) analysis [22].

### 2.5. Identification of Potential Biomarkers Using Untargeted LC-MS/MS Proteomics Analysis

A Dionex 3000 Ultimate nano-LC system (Sunnyvale, CA, USA) connected to an LTQ Orbitrap Velos mass spectrometer (Thermo Scientific, MA, USA), which was equipped with a nano-ESI source, was used for the analysis. LC-MS/MS analysis was conducted on a tryptic digest serum sample corresponding to 1 µg of proteins. Two solvents were used as gradients; 1 is more aqueous, and the 2nd is more organic: mobile phase A was composed of 97.9% HPLC water, 2% can, and 0.1% FA, while mobile phase B was composed of 99.9% ACN and 0.1% FA. Firstly, all 27 samples (Control and NT1 samples) were loaded on the trapping column (Thermo Scientific Acclaim PepMap C18, 75 µm × 20 mm, 3 µm, 100 Å), which helped enrich and desalt the samples (online purification). Then, the samples were further separated with a Thermo Scientific PepMap C18 column (75 µm × 150 mm, 2 µm, 100 Å) with a flow rate of 0.350 µL/min in a 120 min run. Mobile phase B was kept at a slope of 5% for the first 10 min of the run, then increased to 20% over 55 min, 30% over 25 min, 50% over 20 min, and 80% over 1 min, then kept at 5% until the end of the run. The separated peptides were sent into the LTQ Orbitrap Velos mass spectrometer for detection through electrospray ionization.

The mass spectrometer was set at Data Dependent Acquisition (DDA) mode for the two MS scans; the first was an FTMS scan from 400–2000 *m*/*z* and Normalized collision energy (CE) of 35% at a resolution of 60,000 while it worked in positive ion mode. From the MS spectrum, the top 10 most intense ions were selected for fragmentation in the second MS event (MS^2^), and Collision Induced Dissociation (CID) was set as the fragmentation technique with a mass resolution of 7500. The isolation width was 3 *m*/*z*, the dynamic exclusion duration was 90 s, the activation Q was set at 0.250, and the activation time was 10 min.

### 2.6. Untargeted LC-MS/MS Data Analysis

Raw data obtained from the untargeted LC-MS/MS analysis of the 27 serum samples were processed by MaxQuant software, version 1.5.6.4 (Matrix Science Inc., Boston, MA) to attain the peptide intensities when searched against the Swiss Protein human database. The acetylation of the protein N-terminal and the oxidation of methionine were set as variable modifications, and the fixed modification was set for carbamidomethylating of cysteine. The proteomics experiment was conducted on label-free quantification (LFQ); thus, this was set on the MaxQuant, and the LFQ average number of neighbors was set as 6. Peptides were identified at an *m*/*z* tolerance of 6 ppm, while the minimum peptide length was set to 7, with a maximum of two missed cleavages. Finally, the false discovery rate (FDR) was set at 0.01 for identifying peptides and proteins. Further to all these settings, proteins with a minimum of two identified peptides were only considered. In order to visualize the results from the MaxQuant analysis, Perseus software, version 1.5.5.09 (Max Planck Institute of Biochemistry, Munich, Germany), was used, which generated a master file containing all proteins identified in the 27 samples, along with their LFQ intensities. The ion peak intensities represent the relative abundance of the proteins in each sample. The data was further analyzed by GraphPad Prism 9.3.1 (GraphPad Software Company, La Jolla, CA, USA).

Since the data extracted did not satisfy the normality test criteria, a nonparametric test was applied. The levels of identified proteins in the NT1 samples were compared with Control samples using Mann-Whitney *U*-Test. All the identified proteins were compared based on sex differences in the sample, age, and the presence of human leukocyte antigen (HLA). The GenBank ID and gene names of each protein were exported to an excel file to form an experimental data set. Principal Component Analysis (PCA) and heatmap were performed to visualize the differences among different groups. Unsupervised PCA was conducted with OriginPro2022b software (95% confidence level), and a protein-specific heatmap was created with Genesis software version 1.8.1. In addition, to evaluate the predictive ability, selectivity, and sensitivity of each of the DEPs, SPSS^®^ version 28 (IBM) software was used to create receiver operating characteristic curves as well as to obtain the area under curves (AUC) values [23].

### 2.7. Ingenuity Pathway Analysis (IPA) and Gene Ontology for System Biology

Tracking the functions of the DEPs in NT1 in different biological and functional biochemical pathways gives an idea of the possible effect of the DEPs in the pathophysiology of NT1. This could assist in the therapeutic design and drug targeting for the disease. Both pathway studio and IPA software were used for system biology analysis in this study. For the IPA, the gene name of the proteins, *p*-value, as well as the Log2 of the fold change were used for the analysis. Core analysis was performed in a flexible format, and the Gene symbol (human) was set as a platform for the experiment. GenBank or Uniprot/SwissProtein Accession was set as an identity platform for the proteins. According to previous literature, pathways related to NT1 disease were further investigated and scanned to better understand the roles of the significantly expressed proteins in those pathways.

Elsevier’s Pathway Studio v 10.0 (https://www.elsevier.com/solutions/pathway-studio-biological-research (accessed on 23 August 2022)) was employed to establish the relationships between the DEPs and biological processes related to NT1. The validated proteins were designed in hollow color for easy identification and reference. The software generates a proteome-interactome network using a direct-interaction algorithm that maps cellular processes and interactions among genes of the DEPs. Of the 36 proteins, interactions related to the brain and circulation were generated with proteins considered statistically significant after Fisher’s statistical test. This was achieved by the Subnetwork Enrichment Analysis (SNEA) algorithm, which also helps extract statistically significant altered functional pathways. SNEA creates a central seed from all the related entities in the database and retrieves associated entities based on their relationship with the seed—expression targets, regulation targets, and protein modification targets.

The gene ontology (GO) of the DEPs was conducted with “Panther17.0 Released” to understand pathway functions, molecular function, and the effect of those proteins in biological processes (*p*-value cutoff = 0.05). A Hierarchical clustering tree was obtained by online bioinformatic software “Shiny GO v0.75”, which was used to visualize the strength of the relationship between pathways affected by the DEPs [24]. After the quantitative validation of the DEPs, the proteins were processed using the Kyoto Encyclopedia of Genes and Genomes (KEGG) pathway enrichment and another GO, with only the top 10 pathways considered. “Shiny GO v0.75” was also used to predict the biological processes affected by the quantitatively validated DEPs and the proteins’ cellular and molecular functions [24].

### 2.8. Subcellular Localization

After quantitatively validating the proteins, their subcellular distribution was determined by a commonly used prediction software, CELLO v.2.5 [25]. The result was also double-checked with WoLF PSORT, another commonly used subcellular localization software [26]. The software works with different types of sequence coding strategies: the dipeptide composition, partitioned amino acid composition, and the physicochemical properties of the primary structure of the proteins. In addition, WoLF PSORT considers the functional motifs of the proteins. K-nearest neighbor classifier values assigned to the DEPs were then used for prediction.

### 2.9. Targeted Proteomics (LC-PRM-MS) Strategy

A targeted PRM approach was applied to validate some of the DEPs (those with the highest level of fold change and more related to NT1, as predicted by the IPA analysis). First, 1 µL of each of the 27 tryptic digested samples were pooled together, dried, resuspended in the mobile phase, and then run on the Dionex 3000 Ultimate nano-LC system interfaced with a Q-Exactive HF mass spectrometer (Thermo Scientific). Next, 1 µg/µL of the pooled sample was injected into the instrument for the identification of the precursors’ ions, their fragments, as well as retention times. The peptide information of the proteins of interest was identified, collected on Xcalibur, and then used to prepare a transition list for the targeted proteomics. The gradient used for the untargeted proteomics analysis was kept the same.

As usual, after the chromatographic separation, peptides were detected in the Q-Exactive mass spectrometer Orbitrap with the following parameters: run-time 120 min; detection in positive mode; and an MS full-scan range of 200–2000 *m*/*z* followed by MS^2^ scanning at the range of 400–2000 *m*/*z*. A fragmentation pattern of HCD was attempted at normalized collision energy (NCE) 25 and 35 but established at 35 for this analysis. Precursors of the proteins of interest that met the requirements to be considered for a good PRM assay were selected and included in the transition list with a retention time window of ±6 min and a mass range of ±2Da relative to the target mass [27,28]. This was in accordance with an established protocol for targeted proteomics in Yehia Mechref’s Lab [29].

The expected RT of the precursors (obtained from untargeted proteomics) was manually re-checked from the raw data of the pooled sample using Xcalibur (Thermo Scientific) and compared with those in the untargeted proteomics study. Peptides absent in the pooled sample raw data were excluded from the transition list, and 42 peptides were finally targeted, representing 14 DEPs from untargeted proteomics results. For quantitative validation, PRM data was analyzed and quantified by Skyline software version 21.2.0.536, and the normalized data acquired were used for the statistical analysis. The Mann-Whitney *U*-Test was applied to compare the PRM data in NT1 and Control samples using GraphPad Prism 9.3.1 (GraphPad Software Company, La Jolla, CA, USA); precursors with a *p*-value < 0.05 were considered significant.

## 3. Results

### 3.1. Unsupervised PCA for Comparative Proteomics Analysis of NT1 and Control Samples

**Figure 2:**
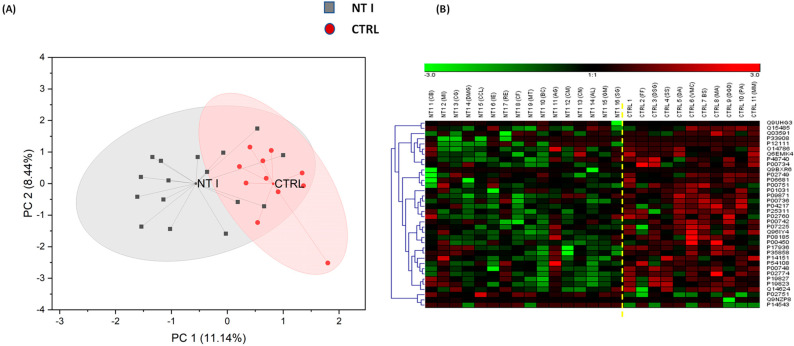
(**A**) Unsupervised Principal Component Analysis (PCA) of all identified proteins at a 95% confidence level and (**B**) Heatmaps for the DEPs in 16 NT1 and 11 control samples. Thirty-two DEPs are downregulated proteins in NT1, indicated by the color green, while four DEPs are upregulated, indicated by the color red.

The combination of MaxQuant version 1.5.6.4 and Perseus 1.6.15.0 identified 195 low-abundant proteins in the 27 depleted serum samples. The unsupervised PCA generated with OriginPro2022b software at a confidence level of 95% for all 195 identified proteins is shown in Figure 2A, and a reasonable level of clustering is observed. The differences in the NT1 and Control sample groups can be seen in the primary principal component 1 (PC1) and the secondary principal component 2 (PC2). Most importantly, this proves that the two groups (NT1 samples and control samples) can be considered as two different cohorts. The NT1 samples are expected to have a different expression level of proteins compared to the healthy control samples. This helps track the proteome as it changes from a healthy state to an NT1 condition in humans.

### 3.2. Heatmap of DEPs

Of the 195 low-abundant proteins, 36 showed statistically significant expression changes (differentially expressed proteins) in a comparison between the NT1 sample group and the control group. The DEPs were used for protein-specific heatmap to visualize their expression levels. Figure 2B shows the protein-specific heatmap, revealing that the proteome of the NT1 serum samples has differentially expressed proteins that can be further investigated. 32 DEPs were observed to be downregulated in NT1, while 4 proteins were upregulated. The 32 proteins have relatively low levels of expression in NT1, while the protein product of the genes FN1, NID, C1RL, and PCYOX are observed to have a higher level of expression in NT1 (Table 2). This is consistent with the calculated fold change of 36 DEPs. The range of visualization is from green (−3.0) to red (3.0), depicting the level of proteome changes between NT1 and healthy samples (control).

### 3.3. Sex and Age-Based Comparison and Volcano Plot

**Figure 3:**
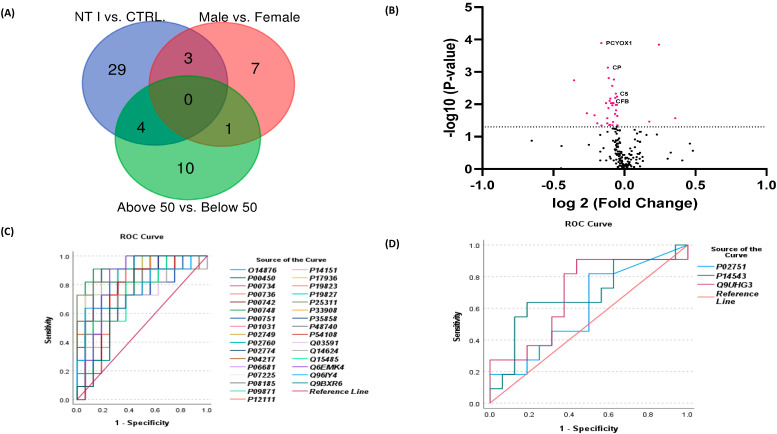
(**A**) Venn diagram of all identified low-abundant proteins in NT1 samples. Protein expression levels are compared based on sex, age, and sample source (disease and control). (**B**) Volcano plot of all identified low-abundant proteins in NT1, proteins with *p*-value < 0.05 upon Mann Whitney *U*-test are considered significant and are colored in magenta. DEPs are above the threshold, with downregulated DEPs on the left and upregulated DEPs on the right. Receiver operating characteristic curve (ROC-AUC) for (**C**) Downregulated DEPs in NT1, the AUC score of the ROC analysis is between the range of 0.80–0.91, while the *p*-value is between the range of 0.0001–0.04. (**D**) In upregulated DEPs in NT1, the AUC score of the ROC analysis is between the range of 0.60–0.70, while the *p*-value is between 0.0001–0.04.

Aside from the 36 DEPs discovered based on the comparison between NT1 samples vs. control, out of 195 identified proteins across all samples, 11 DEPs were determined by sex-based comparison, and 15 DEPs were determined by age-based comparison. The Venn diagram investigating the overlapping proteins is shown in Figure 3A, and the protein content representing the element of each set is given in Table S1. From the gender dimorphism observed in the proteome profiles of NT1 in this study, out of the 11 DEPs, 10 proteins are upregulated in Female NT1 compared to male NT1, while one protein is downregulated in female NT1 compared to Male NT1.

Additionally, the volcano plot shows all the identified proteins, with a threshold line generated at a *p*-value of 0.05 after the Mann–Whitney *U*-test. The data points above the line represent the statistically significant proteins or differentially expressed proteins (DEPs) (Figure 3B) and are painted magenta. The DEPs at the right-hand section of the plot are downregulated in NT1, while the DEPs at the left-hand section of the plot are upregulated. Information about the IDs, *p*-value, FC, and Area Under the Curve (AUC) of the DEPs are shown in Table 2. The down-regulated DEPs were combined; the range of their AUC is 0.73–0.91, with a *p*-value of 0.0001–0.04, whereas the AUC range for the upregulated proteins is 0.45–0.68 when combined (Figure 3C,D). The combined AUC for the downregulated DEPs in NT1 is 0.95, and for the upregulated DEPs in NT1 is 0.76, which indicates that the proteins are relatively selective and a good analyte to differentiate between NT1 and the healthy controls [30]. Because the AUC of Q9NZP8 falls below 0.50, it was not considered for the quantitative validation of DEPs in this study.

### 3.4. Gene Ontology for the Untargeted Proteomics Result

The relationship between the top 30 pathways altered by the DEPs in NT1 is predicted by ShinyGO v0.75, which technically reveals the biological relevance of the 36 DEPs in NT1 (Figure S1A). The gene ontology of the DEPs considering molecular functions and biological functions are also shown in Figure S1B,C, respectively. Essentially, most of the DEPs are involved in the regulation of complement and coagulation cascades (immune response), as well as proteolysis; this is expected because the loss of orexin/hypocretin neuropeptides is well attributed to NT1 [31,32].

### 3.5. PRM Validates DEPs

After the untargeted proteomics study of the NT1, out of the 36 identified DEPs, we targeted 14 proteins that have the highest level of fold change. They are also mostly related to NT1 progression based on the IPA and GO results of the untargeted proteomics analysis (Figures S1 and S2). 11 DEPs were detected by the PRM assay, and eight DEPs were validated by PRM. The eight validated DEPs maintained the same trend of fold change when the untargeted proteomics and the targeted proteomics study of NT1 were carried out. Seven validated proteins were downregulated in NT1 (CP, CFB, C5, ITIH4, AMBP, C2, ITIH2), while one (FN1) was upregulated. As a reference point for comparison, the fold changes of the validated DEPs during targeted and untargeted proteomics studies are provided in Table S1. The table shows the list of the validated DEPs, including their charge state, *m/z*, RT, FC, and *p*-values. 

**Figure 4:**
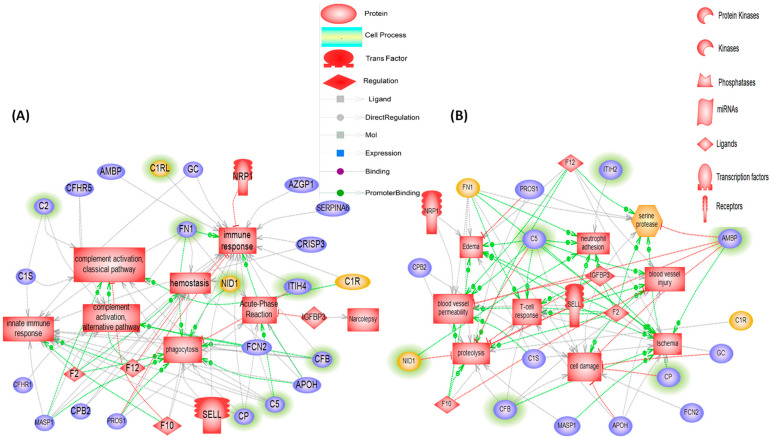
The global interactome of DEPs in NT1. (**A**) Altered pathways and network of DEPs involved, (**B**) Altered cellular processes and network of DEPs associated. The DEPs hollowed in green are validated by LC-PRM-MS.

The pathway studio generated a global interactome of all differentially expressed proteins. The interactome result shows seven altered processes (Figure 4A), including pathways and other biological processes, as well as the network of DEPs that may be associated with NT1. These processes include the complement activation/alternative pathway, complement activation/classical pathway, acute-phase reaction, immune response, hemostasis, innate immune response, and phagocytosis. The interactome also shows eight altered cellular processes and the DEPs involved (Figure 4B), which include neutrophil adhesion, blood vessel injury, T-cell response, proteolysis, cell damage, ischemia, edema, and blood vessel permeability. 

### 3.6. GO, KEGG and Subcellular Localization of the Three Quantitatively Validated DEPs

Among the eight validated DEPs in the targeted proteomics study, three were quantitatively validated and coded by the genes ITIH4, FN1, and C5. The charge states, *m/z*, RT, FC, and *p*-value are given in Table S2, and Figure S3 shows the box plots and ROC curves of the three mentioned proteins. Figure S4A shows the functional and fold enrichment of the three quantitively validated DEPs. The KEGG analysis and GO analysis results for molecular function (Figure S4B), cellular component (Figure S4C), biological function (Figure S4D), and pie-chart of their subcellular distribution (Figure S4E) are also shown. Using GO, we analyzed the top 10 classifications most significantly enriched in cellular and molecular function and biological processes. The functional/fold enrichment analysis predicted that the three quantitatively validated proteins are primarily involved in complement and coagulation cascades, which supports the previously reported information [33]. In addition, the functional/fold enrichment analysis predicted other conditions such as infection of the epithelial cells, AGE-RAGE signaling pathway in diabetic complications, pertussis, and ECM-receptor interaction.

The KEGG and gene ontology enrichment analysis results predicted that the three quantitatively validated proteins are also involved in the acute phase response, regulation of proteolysis and peptidase activity, hydrolase activities (Figure S4D), as well as regulation of important biological processes (Table S3). The loss of orexin/hypocretin neuropeptides in NT1 could be attributed to the dysregulation of proteolysis and peptidases. Essentially, the top 10 cellular components affected by the quantitively validated DEPs include the membrane attack complex and the formation of a fibrinogen complex (Figure S4C). This is also revealed in the IPA result, where a cascade of events in the complement activation pathway is shown. C5 is the first complement protein that constitutes the membrane attack complex that is formed in the pathway (Figure S2A).

Finally, the subcellular localization result predicted that the quantitatively validated DEPs are primarily located in the extracellular and nuclear membrane, but plasma membrane, mitochondria, and cytoplasm were also predicted (Figure S4E).

## 4. Discussion

In this study, we applied the technique of proteome profiling in identifying serum biomarkers specifically for the most common type of narcolepsy (NT1). To our knowledge, this is necessary for improving diagnostic tools for NT1 as it gives the advantage of diagnosing NT1 simply from a serum sample. In addition, we hope this study increases the understanding of the pathogenesis of NT1, which could help in designing therapeutic and prophylactic measures for the disease. Such work is essential for bridging the gaps in the current understanding of narcolepsy in the scientific community [1]. Previous studies have predicted that narcolepsy is associated with autoimmunity after investigating the ‘criteria of autoimmune disease’ in narcolepsy cases [7,34,35,36]. The relationship between human leukocyte antigen (HLA) and NT1 was significantly discussed in a study conducted by Giannoccaro et al. [36]. Thus, we investigated the presence of HLA in the patients’ samples selected for this study. Out of 16 narcolepsy patients, 15 tested positive for HLA, and one did not. We further identified 21 differentially expressed proteins by comparing HLA presence in our samples.

In most cases, the expression of low-abundant proteins in serum and the alterations in their expressions are associated with a change in the organism’s physiological state. However, due to the wide dynamic range of serum proteins, the presence of low-abundant proteins is often masked by the high-abundant serum proteins, thereby affecting the detection of the low-abundant proteins that are currently considered potential biomarkers of diseases [37,38,39]. Hence, in this study, we focused on the low-abundant proteins by depleting the samples off the high-abundant proteins prior to the proteome profiling of the samples. When we compared the NT1 and healthy control groups, eventually, we found some similarities and many differences in the proteomes of the two groups. When the two groups were compared, 36 unique proteins were significantly expressed, and 32 of those were downregulated in NT1. Among the DEPs, the protein products of the genes C1RL, NID1, FN1, and PCYOX were upregulated in NT1. C1RL was previously found to be involved in immunological activities and reported as an independent prognostic biomarker in glioblastoma (GBM); interestingly, C1RL was also found to be upregulated (in GBM) in the study [40,41]. FN1 is a gene commonly reported in gene expression studies related to cancer and neurodegenerative diseases [19,42,43]. The synergetic effect of FN1 and genes of some DEPs in NT1 might have resulted in the dysregulation of neutrophils and leukocytes and might contribute to the complex relationship between orexin and cancer or neurodegeneration that has recently been indicated as a possible case of inverse comorbidity [44]. The function of PCYOX, which was previously unrecognized, was recently found and reported to be involved in controlling neutrophil Rhebs levels, thereby affecting neutrophil bactericidal potential [45].

Increased obesity and type-2 diabetes due to insulin resistance in the orexin deficiency state of narcolepsy patients have been reported in prior studies [4,46,47]. Excitingly, our KEGG functional enrichment analysis in this study predicted the effect of the quantitatively validated DEPs in the AGE-RAGE signaling pathway in diabetic complications and bacterial invasion of epithelial cells (Figure S4A), which further confirms the relationship between diabetes and narcolepsy. Furthermore, the Mignot group from Stanford University previously reported that infection could be the highest environmental risk for narcolepsy [48]. FN1, C5, and ITIH4 are all quantitatively validated. The summary of the functions of DEPs in this study can be understood from the results of the gene ontology analysis (Figure S1). In a study that investigated the alterations of classical component pathway factors in narcolepsy, C1Q was reported to be upregulated; it was also found to be upregulated in our study but did not meet the criteria to be statistically significant [33]. Some other complement proteins identified in these studies are found to be downregulated. Also, ITIH3 and PTPRN2 are proteins found in this study and have also been reported in proteomics profiling in narcolepsy rat models, as well as some other gene expression studies [19].

The acute phase response signaling pathway explains the biochemistry behind the cascade of events leading to the inflammatory response that confers protection of the body against infections. This signaling pathway is also triggered by tissue injury and acute-phase cytokines, and it has been previously reported that the acute phase response leads to changes in the body system, including sleepiness (excessive), which is a major symptom of narcolepsy; anorexia; and protein production, especially the complement proteins [49]. Interestingly, six of the DEPs identified by this study are involved in the complement activation pathway, coded by gene C2, CFB, C5, C1R, C1S, and MASP1; and eleven are involved in Acute Phase Response Signaling (APRS): FN1, AMBP, APOH, CFB, CP, ITIH2, C5, C2, F2, C1, and ITIH4.

Notably, the integrin signaling pathway, p53 pathways, and axon guidance mediated by ‘semaphorins’ revealed by GO analysis are understandable as this may support the relationship between signaling pathways and inflammatory regulation as well as immune response mediation [50]. The p53 pathway has previously been reported to play a pivotal role in neurodegenerative diseases [51]. Figure S5 shows the interconnection of processes affected by the three quantitatively validated proteins.

In conclusion, autoimmunity does not necessarily have to be confirmed by upregulation of all complement proteins but rather complement function dysfunction, which could be upregulation, downregulation, or dysfunction of complement proteins, as all of these can synergistically lead to autoimmunity; this aligns with a recent review study that explored the relationship between the complement system and autoimmunity [52]. Hence, to the best of our knowledge, our study confirms the relationship between autoimmunity and NT1 and explains some insights regarding the involved bioprocesses.

However, it is important to stress the concept that this proteomic study most probably reflects the combined effect of genetic and epigenetic factors, which definitely interact with the pathophysiological mechanisms of NT1 [53]. For this reason, it is important to underline the high AUC found with the combined assembly of underregulated proteins, which seems to indicate that a complex proteomic pattern can be more helpful than single proteins in the identification of NT1 patients and in the characterization of their dysfunctional pathways.

## Figures and Tables

**Table 1:** Specifications of human study participants.

Group Information	Narcolepsy Type 1	Controls
Sample number	16	11
Sex	11 males, 5 females	7 males, 4 females
Age range (years)	19–71	28–73
HLA DQB1*0602 allele	15 samples	2 Samples
Cerebrospinal Fluid Orexin, pg/mL	0–119.6 (Undetectable in 6 patients, not available in 3 patients)	Not available
Body Mass Index	20.2–28.6	Not available

**Table 2:** Up- and down-regulated DEPs in NT1.

Protein Accession	Gene Name	*p*-Value	Fold Change	ROC/AUC
Downregulated Proteins				Combined AUC = 0.95
O14786	NRP1	0.0001	0.83	0.80
P00450	CP	0.0002	0.82	0.80
P00734	F2	0.0008	0.86	0.81
P00736	C1R	0.001	0.88	0.81
P00742	F10	0.002	0.80	1.0
P00748	F12	0.002	0.68	0.91
P00751	CFB	0.003	0.83	0.74
P01031	C5	0.005	0.87	0.77
P02749	APOH	0.006	0.77	0.76
P02760	AMBP	0.007	0.89	0.76
P02774	GC	0.008	0.76	0.87
P04217	A1BG	0.009	0.86	0.87
P06681	C2	0.009	0.82	0.81
P07225	PROS	0.009	0.79	0.81
P08185	CBG	0.009	0.44	0.85
P09871	C1S	0.009	0.84	0.85
P12111	COL6A3	0.010	0.54	0.77
P14151	SELL	0.010	0.75	0.74
P17936	IGFBP3	0.009	0.74	0.78
P19823	ITIH2	0.01	0.88	0.79
P19827	ITIH1	0.02	0.88	0.82
P25311	AZGP1	0.02	0.77	0.85
P33908	MAN1A1	0.02	0.61	0.76
P35858	IGFALS (ALS)	0.03	0.75	0.76
P48740	MASP1	0.03	0.78	0.80
P54108	CRIS3	0.03	0.82	0.73
Q03591	CFHR1	0.04	0.64	0.74
Q14624	ITIH4	0.04	0.89	0.73
Q15485	FCN2	0.04	0.78	0.73
Q6EMK4	VASN	0.04	0.81	0.84
Q96IY4	CPB2	0.04	0.85	0.77
Q9BXR6	CFHR5	0.04	0.79	0.73
Upregulated Proteins				Combined AUC = 0.76
Q9NZP8	C1RL	0.04	1.08	0.45
P14543	NID1	0.03	2.27	0.60
P02751	FN1	0.006	1.61	0.69
Q9UHG3	PCYOX	0.0001	1.49	0.68

## Data Availability

The mass spectrometry proteomics data have been deposited to the ProteomeXchange Consortium via the PRIDE partner repository with the dataset identifier PXD038248 and 10.6019/PXD038248. All the information necessary to access the data is provided using the following credentials: Username: reviewer_pxd038248@ebi.ac.uk; Password: zDbnEmUu.

## Supplementary Materials

The following supporting information can be downloaded at: https://www.mdpi.com/article/10.3390/biom13030420/s1, Table S1: A table summarizing data represented in the Venn diagram that compares the DEPs discovered upon the comparison of identified proteins based on age, sex, and sample source; Table S2: List of proteins of peptides validated by LC-PRM-MS including z, *m*/*z*, RT, FC before validation and FC after validation by PRM. Q14624, P01031, and P02751 are the quantitatively validated proteins when Mann-Whitney *U*-test was applied. Their *p*-values are 0.03, 0.24, and 0.01, respectively; Table S3: High-level GO analysis of the three quantitatively validated proteins. Their specific functions and the genes involved; Figure S1: (A) Hierarchal clustering of the biological significance of pre-validated DEPs and summary of the relationship between the top 30 pathways that the DEPs alter. Bigger dots indicate more significant *p*-values. GO of all DEPs with respect to (B) molecular functions and (C) biological functions; Figure S2: Ingenuity Pathway Analysis of the two major pathways associated with DEPs in NT1. (A) Complement Activation Pathway (B) Acute Phase Response Signaling Pathway. The red color denotes the upregulation of proteins, and the green color indicates the downregulation of proteins in NT1 when compared to the control. Proteins enclosed in a square and a rhombus shape are among the DEPs; Figure S3: Boxplot and ROC curve of the quantitatively validated DEPs coded by genes (A) FN1 (C) C5 (C) ITIH4. After Mann-Whitney *U*-test, nonsignificant (ns) = *p* > 0.05; * = *p* < 0.05; ** = *p* < 0.01; *** *p* < 0.001; Figure S4: KEGG analysis of the three quantitatively validated DEPs based on reactome pathway annotation (A) Functional and fold enrichment analysis. High-level gene ontology for (B) Molecular function, (C) Cellular function, and (D) biological function. (E) Subcellular distribution of the three quantitatively validated DEPs. In all, only the top 10 pathways are considered; Figure S5: Interconnection of the top 10 biological pathways affected by the three quantitatively validated DEPs generated by Shiny GO v7.5. The green dots are the major connection points, and their color intensities indicate the level of the effect of the three proteins on the processes.

## Abbreviations

ABC	ammonium bicarbonate
ACN	acetonitrile
NT1	narcolepsy type 1
FDR	false discovery rate
DTT	dithiothreitol
HLA	human leukocyte antigen
FA	formic acid
IAA	iodoacetamide
IPA	ingenuity pathway analysis
DEPs	differentially expressed proteins
LFQP	label-free quantitation proteomics
PCA	principal component analysis
HPLC	high-performance liquid chromatography
KEGG	Kyoto encylopedia of genes and genomes
