# Supplementary material for: LC-MS/MS-Based Proteomics Approach for the Identification of Candidate Serum Biomarkers in Patients with Narcolepsy Type 1
Source: Biomolecules. 2023 Feb 23;13(3):420. doi: 10.3390/biom13030420 (PMC10046664; doi:10.3390/biom13030420)
Supplement: Supplementary file 1 [file biomolecules-13-00420-s001.zip › biomolecules-2197047-supplementary.pdf]

SUPPORTING INFORMATION: LC-MS/MS-based Proteomics Approach for the Identification of Candidate Serum Biomarkers in Patients with Narcolepsy Type 1

Akeem Sanni 1 , Mona Goli 1, Jingfu Zhao 1, Junyao Wang 1 , Chloe Barsa 2, Samer El Hayek 3 , Farid Talih 4,
Bartolo Lanuzza 5, Firas Kobeissy 2,6 , Giuseppe Plazzi 7,8, Monica Moresco 7, Stefania Mondello 9 ,
Raffaele Ferri 5 and Yehia Mechref 1,*

1 Chemistry and Biochemistry Department, Texas Tech University, Lubbock, TX 79409-1061, USA
2 Faculty of Biochemistry and Molecular Genetics, American University of Beirut, Beirut 1107 2020, Lebanon
3 Department of Psychiatry and Behavioral Sciences, University of Miami Miller School of Medicine, Miami, FL 33124, USA
4 Department of Psychiatry, Faculty of Medicine, American University of Beirut, Beirut 1107 2020, Lebanon
5 Sleep Research Centre, Department of Neurology IC, Oasi Research Institute-IRCCS, 94018 Troina, Italy
6 Multiomics & Biomarkers, Department of Neurobiology, Center for Neurotrauma, Morehouse School of Medicine (MSM), Atlanta, GA 30310, USA
7 IRCCS, Instituto delle Scienze Neurologiche di Bologna, 40139 Bologna, Italy
8 Department of Biomedical, Metabolic and Neural Sciences, University of Modena and Reggio Emilia, 41125 Modena, Italy
9 Department of Biomedical and Dental Sciences and Morphofunctional Imaging, University of Messina, 98122 Messina, Italy
* Correspondence: yehia.mechref@ttu.edu; Tel.: +1-806-834-8246

Supporting Information Table S1: A table summarizing the represented in the Venn diagram that compares the DEPs discovered upon the comparison of all identified proteins based on age, gender, and sample source.

Names	Total	Elements
Male NT1 *vs.* Female NT1 (Gender)	3	A1BG, FHR5, C1RL
Above age 50 NT1 *vs.* Below age 50 NT1 (Age)	4	C5, PROS, ITIH1, ALS
Gender *vs.* Age	1	A2GL
NT1 *vs.* CTRL<br>(Not overlapped)	29	C1S, C1R, VASN, APOH, MASP1, ITIH2, PCYOX, CPB2, CFHR1, CFB, F10, ITIH4, COL6A3, MAN1A1, NRP1, NID1, F12, AMBP, FCN2, AZGP1, SELL, IGFBP3, SERPINA6,<br>GC, CP, CRISP3, F2, FN1, C2
Male *vs.* Female<br>(Not overlapped)	7	PZP, ADIPOQ, PTPRJ, SHBG, CD14, FBLN3, C6
Above age 50 *vs.* Below age 50 (Not overlapped)	10	ITIH3, AHSG, PTGDS, SPRC, PLF4, C4A, APOF, IGF1, C9, SERPINA4

Supporting Information Table S2: List of proteins of peptides validated by LC-PRM-MS including z, *m*/*z*, RT, FC before validation and FC after validation by PRM. Q14624, P01031, and P02751 are the quantitatively validated proteins when Mann-Whitney *U*-test was applied. Their *p*-values are 0.03, 0.24, and 0.01 respectively.

Protein Accession	Gene name	Annotated sequence	z	m/z	RT	FC	FC (PRM)
**Q14624**	**ITIH4**	**DQFNLIVFSTEATQWRPSLVPASAENVNK**	**3**	**1087.891**	**98.3**	**0.55**	**0.89**
P00450	CP	GPEEEHLGILGPVIWAEVGDTIR	2	1244.148	98.0	0.59	0.82
P00751	CFB	RDLEIEVVLFHPNYNINGK	3	757.404	78.3	0.63	0.83
**P01031**	**C5**	**LLVYYIVTGEQTAELVSDSVWLNIEEK**	**2**	**1556.313**	**32.5**	**0.24**	**0.87**
**P02751**	**FN1**	**DTLTSRPAQGVVTTLENVSPPR**	**3**	**780.084**	**61.1**	**3.66**	**1.61**
P19823	ITIH2	AGELEVFNGYFVHFFAPDNLDPIPK	3	946.137	103.5	0.72	0.88
P00450	CP	ALYLQYTDETFR	2	760.377	62.5	0.65	0.82
P02751	FN1	DDKESVPISDTIIPAVPPPTDLR	3	825.771	73.7	2.54	1.61
P06681	C2	KNQGILEFYGDDIALLK	3	646.351	89.6	0.49	0.81

Supporting Information Table S3: High-level GO analysis of the three quantitatively validated proteins. Their specific functions and the genes involved.

Number of proteins involved	High-level GO category	Genes
3	Response to stress	ITIH4, C5, FN1
3	Regulation of molecular function	ITIH4, C5, FN1
2	Immune system process	C5, FN1
2	Locomotion	C5, FN1
2	Anatomical structure morphogenesis	C5, FN1
2	Regulation of localization	C5, FN1
2	Regulation of locomotion	C5, FN1
2	Regulation of locomotion	C5, FN1
2	Biological process involved in interspecies interaction between organisms	C5, FN1
2	Regulation of response to stimulus	C5, FN1
2	Anatomical structure formation involved in morphogenesis	C5, FN1
2	Cell motility	C5, FN1
2	Regulation of developmental process	C5, FN1
2	Leukocyte migration	C5, FN1
2	Regulation of multicellular organismal process	C5, FN1
2	Cellular localization	ITIH4, FN1
2	Localization of cell	C5, FN1
1	Biological adhesion	FN1
1	Growth	FN1
1	Immune effector process	C5
1	Activation of immune response	C5
1	Regulation of immune system process	C5
1	Immune response	C5
1	Cell adhesion	FN1
1	Cell population proliferation	FN1
1	Response to external stimulus	C5
1	Response to biotic stimulus	C5
1	Cell growth	FN1
1	Cytolysis	C5
1	Regulation of signaling	FN1
1	Regulation of cell adhesion	FN1
1	Regulation of growth	FN1
1	Taxis	C5

**1**	Cellular component biogenesis	FN1
**1**	Developmental growth	FN1
**1**	Response to other organisms	C5
**1**	Protein activation cascade	FN1

Supporting Information Figure 1

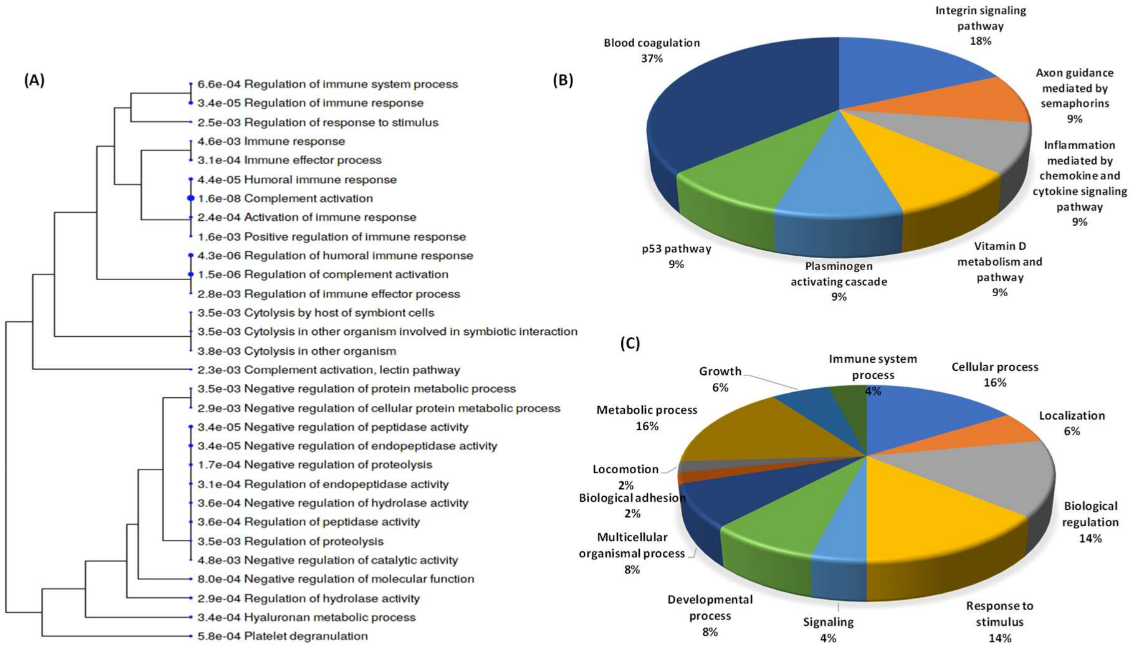

Supporting Information Figure S1: (A) Hierarchal clustering of the biological significance of pre-validated DEPs and summary of the relationship between the top 30 pathways that the DEPs alter. Bigger dots indicate more significant *p*-values. GO of all DEPs with respect to (B) molecular functions and (C) biological functions.

Supporting Information Figure 2

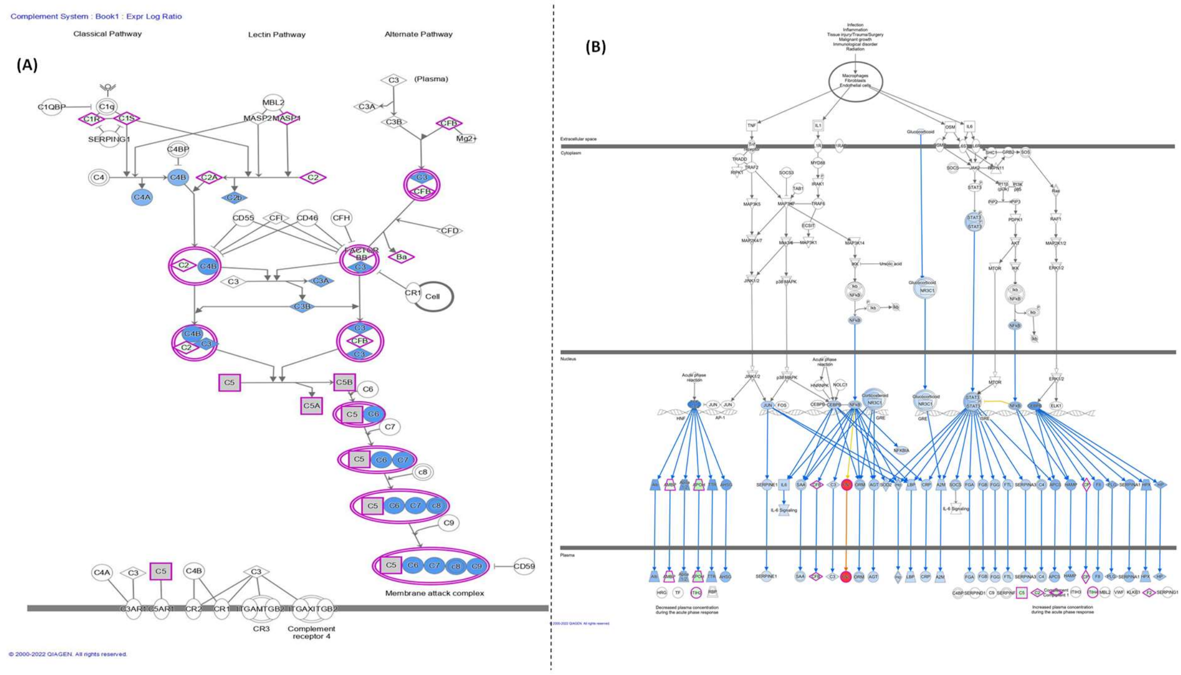

Supporting Information Figure S2: Ingenuity Pathway Analysis of the two major pathways associated with DEPs in NT1. (A) Complement Activation Pathway (B) Acute Phase Response Signaling Pathway. The red color denotes the upregulation of proteins, and the green color indicates the downregulation of proteins, in NT1 when compared to the Control. Proteins enclosed in a square and a rhombus shape are among the DEPs.

Supporting Information Figure S3

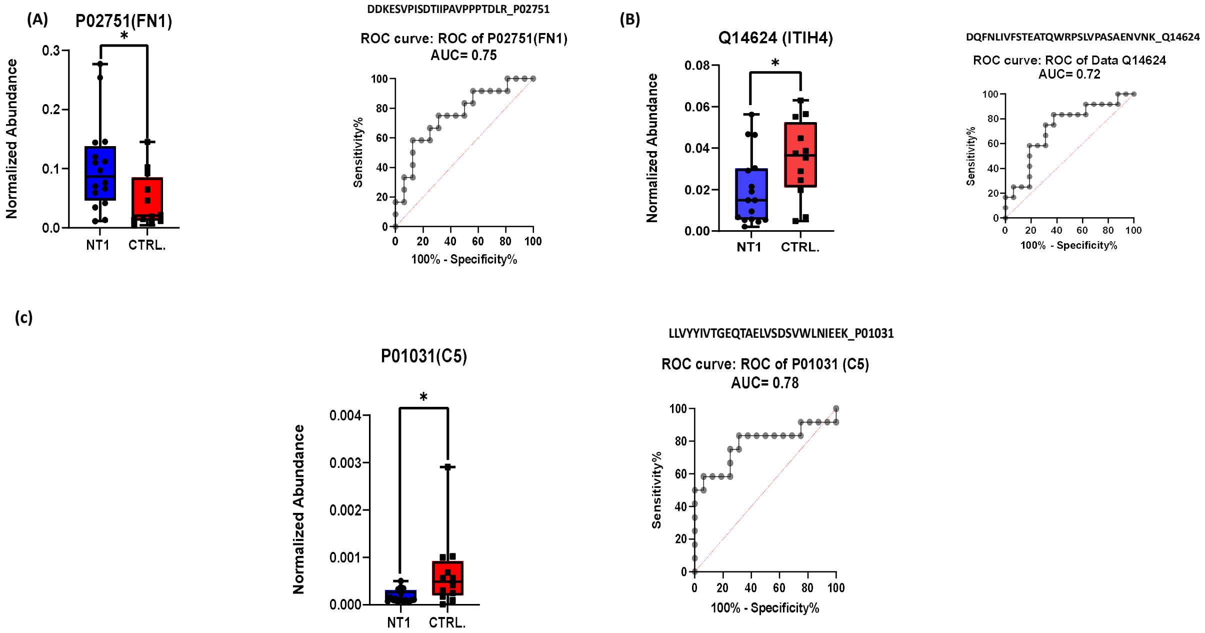

Supporting Information Figure S3: Boxplot and ROC curve of the quantitatively validated DEPs coded by genes (A) FN1 (C) C5 (C) ITIH4. After Mann-Whitney *U*-test, Nonsignificant (ns)= $p > 0.05$; *= $p < 0.05$; **= $p < 0.01$; *** $p < 0.001$

Supporting Information Figure S4

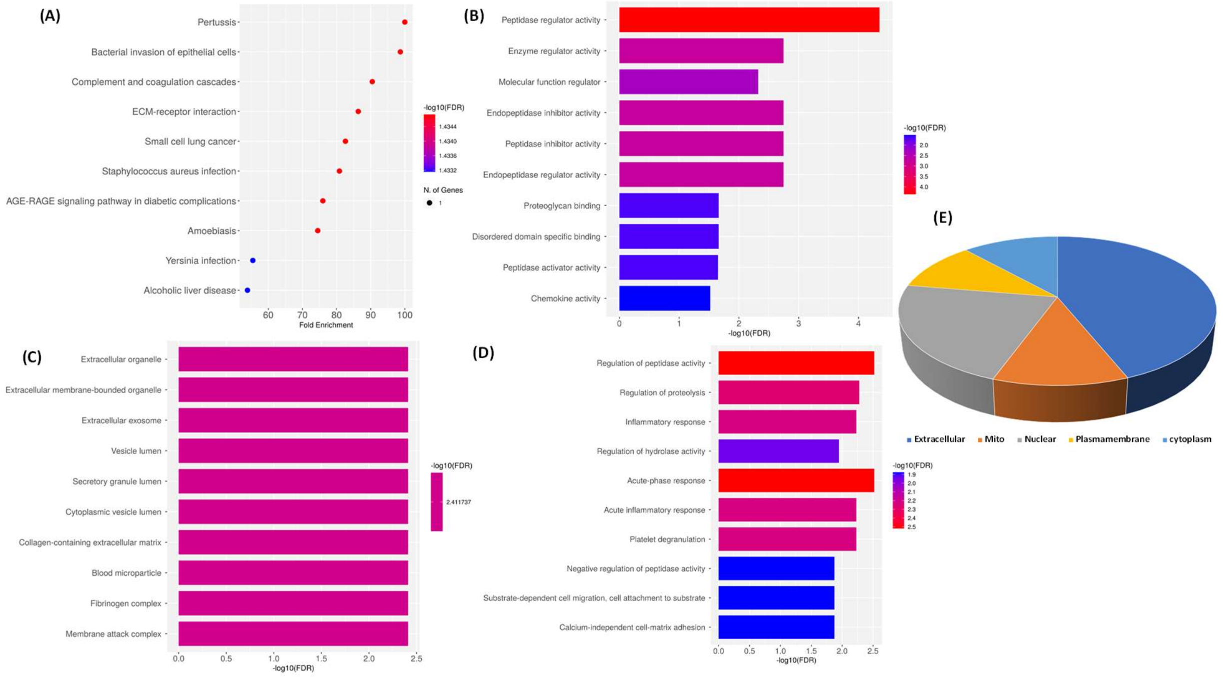

Supporting Information Figure S4: KEGG analysis of the three quantitatively validated DEPs based on reactome pathway annotation (A) Functional and fold enrichment analysis. High-level gene ontology for (B) Molecular function, and (C) Cellular function, and (D) biological function. (E) Subcellular distribution of the three quantitatively validated DEPs. In all, only top 10 pathways are considered.

Supporting Information Figure S5

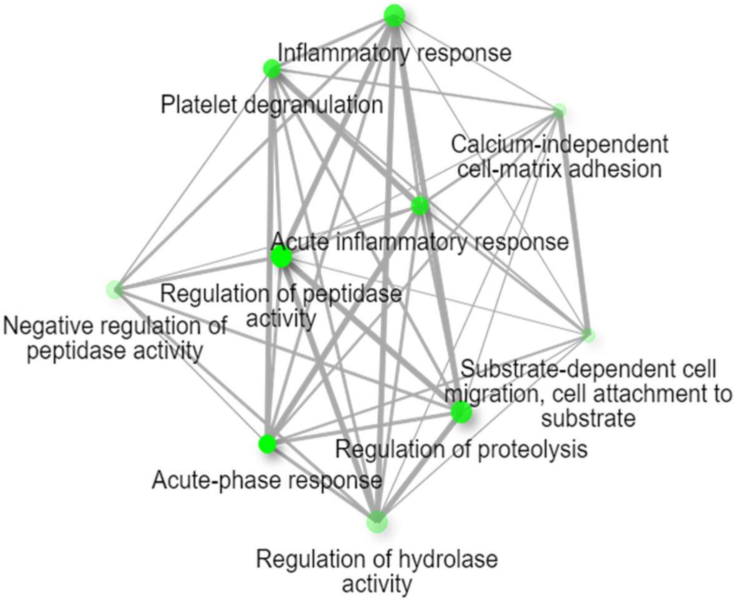

Supporting Information Figure S5: Interconnection of the top 10 biological pathways affected by the three quantitatively validated DEPs generated by Shiny GO v7.5. The green dots are the major connection points, and their color intensities indicate the level of the effect of the three proteins on the processes.
